# Optimal angle of magnetic field for magnetic bubblecade motion

**DOI:** 10.1038/s41598-017-03832-4

**Published:** 2017-06-16

**Authors:** Duck-Ho Kim, Kyoung-Woong Moon, Sang-Cheol Yoo, Dae-Yun Kim, Byoung-Chul Min, Chanyong Hwang, Sug-Bong Choe

**Affiliations:** 1 Department of Physics and Institute of Applied Physics, Seoul National University, Seoul, 08826 Republic of Korea; 2 Center for Nanometrology, Korea Research Institute of Standards and Science, Daejeon, 34113 Republic of Korea; 3 Center for Spintronics, Korea Institute of Science and Technology, Seoul, 02792 Republic of Korea; 4 Institute for Chemical Research, Kyoto University, Uji, Kyoto 611-0011 Japan

## Abstract

Unidirectional motion of magnetic structures such as the magnetic domain and domain walls is a key concept underlying next-generation memory and logic devices. As a potential candidate of such unidirectional motion, it has been recently demonstrated that the magnetic bubblecade—the coherent unidirectional motion of magnetic bubbles—can be generated by applying an alternating magnetic field. Here we report the optimal configuration of applied magnetic field for the magnetic bubblecade. The tilted alternating magnetic field induces asymmetric expansion and shrinkage of the magnetic bubbles under the influence of the Dzyaloshinskii-Moriya interaction, resulting in continuous shift of the bubbles in time. By examining the magnetic bubblecade in Pt/Co/Pt films, we find that the bubblecade speed is sensitive to the tilt angle with a maximum at an angle, which can be explained well by a simple analytical form within the context of the domain-wall creep theory. A simplified analytic formula for the angle for maximum speed is then given as a function of the amplitude of the alternating magnetic field. The present results provide a useful guideline of optimal design for magnetic bubblecade memory and logic devices.

## Introduction

Magnetic domain-wall (DW) motion has been intensively studied as a test body of the emerging spin-dependent phenomena^1–5^ as well as a building block of the potential memory and logic devices^6–9^. Such DW motion has been achieved by the spin-orbit^2–4, 10^ or spin-transfer^11–14^ torques through injection of the spin-polarized current for realization of the DW-based racetrack memory^4, 6^. Fairly recently, Moon *et al*.^9^ proposed another scheme to generate a similar motion by applying an alternating magnetic field to chiral DWs. The coherent unidirectional bubble motions generated by this scheme is referred as a “magnetic bubblecade”, which enables the demonstration of multi-bit bubble memory operation. The key concept underlying this scheme relies on the broken symmetry and chiral DW formation caused by the Dzyaloshinskii–Moriya interaction (DMI), which induces the asymmetric expansion and shrinkage of magnetic bubbles^9, 15, 16^.

The present chiral magnetic bubbles have the topological structure similar to the magnetic skyrmions except the size and therefore, a similar motion can be achieved from the magnetic skyrmions^17, 18^, which are useful for better data storage density. The magnetic bubblecade memory is similar to the magnetic racetrack memory^6^, but the operation principles are totally different: the magnetic bubblecade is operated by applying an alternating mangetic field, whereas the magnetic racetrack is operated by injecting electric current into elaborated nanometer-sized wire patterns. It is also worthwhile to compare the magnetic bubblecade memory with the magnetic bubble memory^19^ commercialized in 1970s, which is operated by attracting and repelling the bubble domains along the tiny magnetic guide patterns under rotating magnetic field. The present magnetic bubblecade memory is relatively free from such geometric restrictions essential for the racetrack and magnetic bubble memories.

Here, we investigate the optimal angle and magnitude of the external alternating magnetic field for the magnetic bubblecade. For this study, the magnetic bubblecade is realized in Pt/Co/Pt films with sizable DMI^15^, which have a strong perpendicular magnetic anisotropy (PMA)^20, 21^. The bubblecade speed is then examined with respect to the tilt angle and magnitude of the external alternating magnetic field. A clear angular dependence is observed and explained using DW creep theory, which provides an optimal design rule for the magnetic bubblecade.

## Results

### Schematic Diagram and Experimental Observation of the Bubble Motion

**Figure 1:**
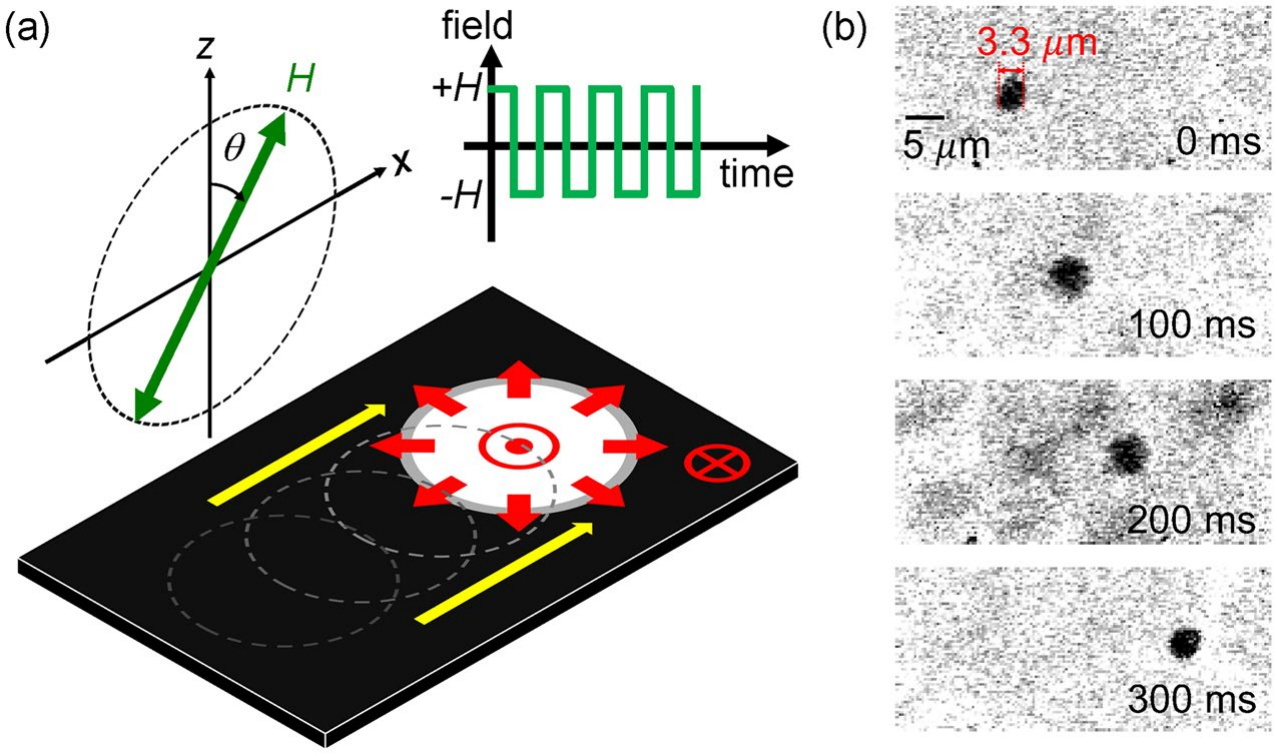
Schematic descriptions of the magnetic bubblecade induced by tilted alternating magnetic field. (**a**) Illustration of a bubble domain (bright circle) and the DW (grey ring), surrounded by a domain of opposite magnetization (dark area). The red symbols and arrows indicate the direction of the magnetization inside the DW and domains. The dashed circles represent the previous bubble positions and the yellow arrow indicates the direction of the bubble motion. (**b**) Experimental observation of the unidirectional bubble motion with applying magnetic field pulses (${\mu }_{0}H=\,$65.8 mT, $\theta \,=\,$81.${2}^{^\circ }$) taken after application pulses (each time means 2 $\Delta t$), where black areas indicate up domains surrounded by a opposite magnetization (white areas).

Figure 1(a) shows a magnetic bubble (up domain) with the Néel DW configuration caused by a positive DMI^15, 16, 22^. The magnetization (red arrows) inside the DW is pointing radially outward in all directions. By applying a tilted alternating magnetic field, a bubblecade along the +*x* direction (yellow arrow) was generated^9^, of which the speed *ν* was measured with respect to the tilt angle *θ* and the amplitude *H* of the alternating magnetic field by use of a magneto-optical Kerr effect (MOKE) microscope. The tilt angle *θ* of the electromagnet is defined from the +*z* direction to the +*x* direction is shown in Fig. 1. Figure 1(b) presents images of a unidirectional bubble motion captured by the MOKE microscope.

### Angle Dependence of Bubble Motion

**Figure 2:**
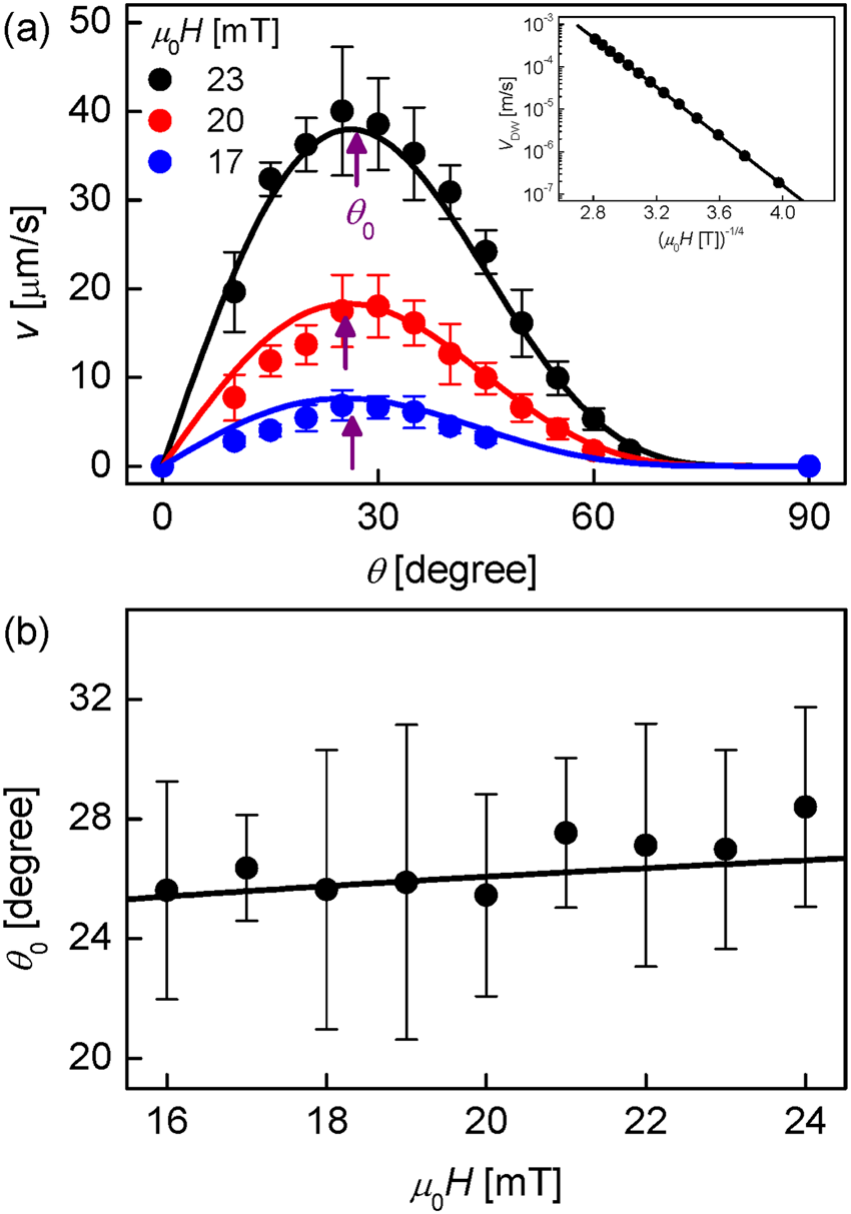
Angle dependence of magnetic bubble motion. (**a**) Measured $v$ with respect to *θ* for several *H* (symbols). The solid lines are best fits with Eq. (1). The purple arrow represents *θ*_0_. The inset plots the DW speed ${V}_{{\rm{DW}}}$ (i.e. the bubble expansion speed) with respect to the purely out-of-plane magnetic field (i.e. *θ* = 0). The error bars are obtained by the standard deviation of more than 20 times repeated measurements. (**b**) *θ*_0_ with respect to *H*. The solid line is the numerical evaluation of Eq. (3) with the experimental value of *α*_0_ ( = 6.7 T^1/4^). The error bars are obtained by the root-mean-square error of the parabolic fitting.

Figure 2(a) plots the measured $v$ with respect to *θ* under several fixed *H* as denoted inside the plot. It is clear from the figure that each $v$ curve exhibits a maximum at an angle *θ*_0_ as indicated by the purple arrow. Hereafter, *θ*_0_ will denote the angle for the maximum $v$. The measured *θ*_0_ is plotted with respect to *H* in Fig. 2(b). The inset of Fig. 2(a) shows that the DW speed ${V}_{{\rm{DW}}}$ under application of purely out-of-plane magnetic field (i.e. $\theta =0$) exactly follows the DW creep criticality by showing the linear dependence with respect to ${H}^{-1/4}$.

According to ref. 9, $v$ is defined as $[{V}_{{\rm{DW}}}(+{H}_{z},+{H}_{x})+{V}_{{\rm{DW}}}(-{H}_{z},-{H}_{x})]$ under alternating squared magnetic field between $(+{H}_{z},+{H}_{x})$ and $(-{H}_{z},-{H}_{x})$. Since the effect of ${H}_{x}$ is quite smaller than that of ${H}_{z}$ ^15^, the Taylor series expansion gives a good approximated expression as $v\cong {\gamma }_{1}({H}_{z}){H}_{x}$, where ${\gamma }_{1}({H}_{z})={\partial {V}_{{\rm{DW}}}/\partial {H}_{x}|}_{{H}_{x}=0}$. In the creep regime. In the creep regime, the DW speed follows the creep criticality i.e. ${V}_{{\rm{DW}}}({H}_{z},{H}_{x})={V}_{0}\,\exp [-\alpha ({H}_{x}){({\mu }_{0}{H}_{z})}^{-1/4}]$. Then, the bubble speed can be rewritten as $v=\beta {\mu }_{0}{H}_{x}{({\mu }_{0}{H}_{z})}^{-1/4}\,\exp [-{\alpha }_{0}{({\mu }_{0}{H}_{z})}^{-1/4}]$, where *β* is a constant related to the asymmetry in the DW motion and *α*_0_ is the creep scaling constant for ${\mu }_{0}{H}_{x}=0$. The validity of the present formula was confirmed by experiment for the range of ${\mu }_{0}{H}_{x}$ smaller than 50 mT. By replacing the strengths of the magnetic field as ${\mu }_{0}{H}_{z}\equiv {\mu }_{0}H\,\cos \,\theta$ and ${\mu }_{0}{H}_{x}\equiv {\mu }_{0}H\,\sin \,\theta$, the equation can be rewritten as a function of *H* and *θ* as given by

1 $$v(\theta ,H)=\beta {({\mu }_{0}H)}^{3/4}\,\sin \,\theta {(\cos \theta )}^{-1/4}\exp [-{\alpha }_{0}{({\mu }_{0}H)}^{-1/4}{(\cos \theta )}^{-1/4}].$$

The solid lines in Fig. 2(a) show the best fits with Eq. (1). In this fitting, the experimental value of ${\alpha }_{0}$ (=6.7 T^1/4^) is used, which was determined from an independent measurement of the DW creep criticality^20, 21, 23–26^. Therefore, the fitting was done with a single fitting parameter *β*. The good conformity supports the validity of the present equation.

For a given *H*, *θ*_0_ can be obtained from the maximization condition with respect to *θ* i.e. ${\partial v/\partial \theta |}_{\theta ={\theta }_{0}}=0$, as

2 $${(\cos {\theta }_{0})}^{1/4}(4\,{\cot }^{2}{\theta }_{0}+1)={\alpha }_{0}{({\mu }_{0}H)}^{-1/4}.$$

**Figure 3:**
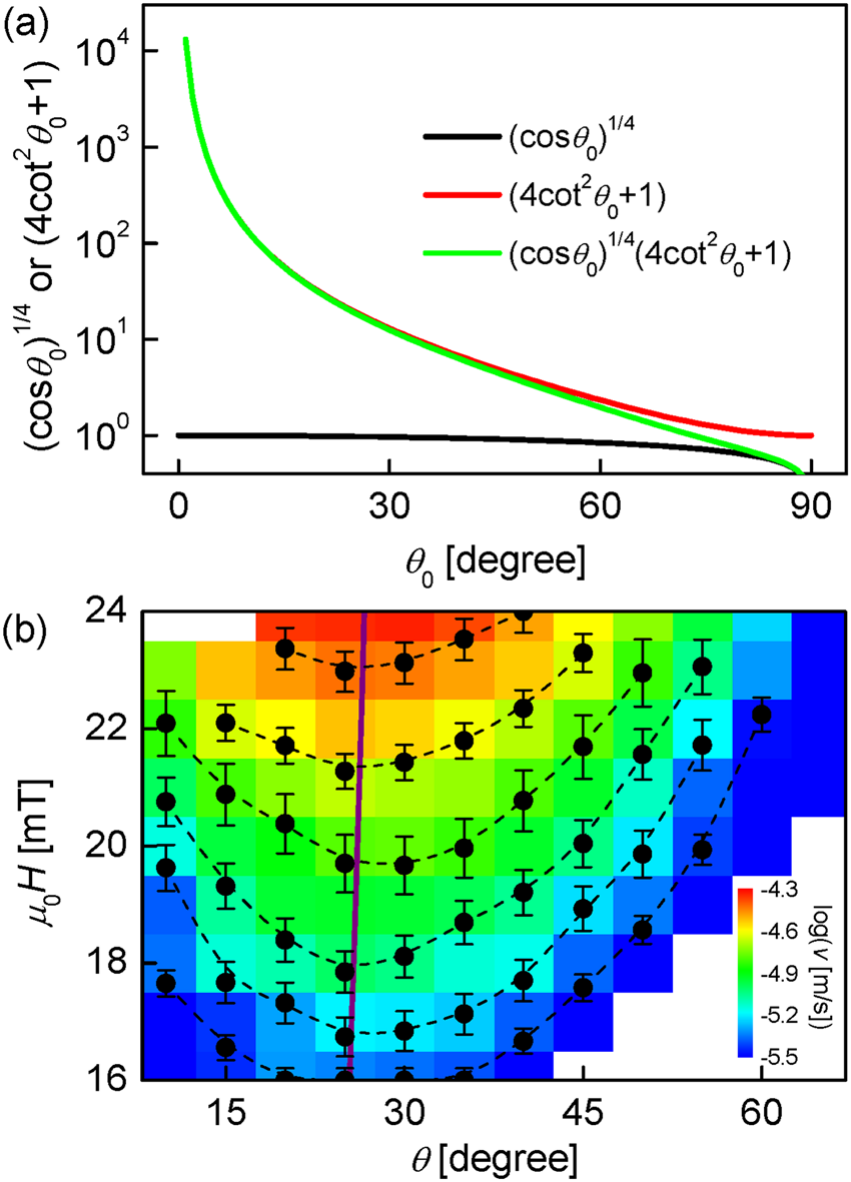
Simplification of ${(\cos {{\theta }}_{0})}^{1/4}(4\,{\cot }^{2}{{\theta }}_{0}+1)$ and two-dimensional contour map of ${v}({\theta },{H})$ as a function of ***θ*** and *H*. (**a**) Numerical calculations of ${(\cos {\theta }_{0})}^{1/4}$, $(4\,{\cot }^{2}{\theta }_{0}+1)$, and ${(\cos {\theta }_{0})}^{1/4}(\,4\,{\cot }^{2}{\theta }_{0}+1)$ as a function of *θ*_0_. (**b**) Two-dimensional contour map of $v(\theta ,H)$ plotted with respect to *θ* and *H*. The colour contrast represents the value of $\mathrm{log}(v)$ with scale bar on the right lower end. The purple solid line is the numerical evaluation of Eq. (3). The black dashed lines guide the eyes to the equi-speed contours.

Since the variation of ${(\cos {\theta }_{0})}^{1/4}$ is negligibly small in comparison to $(4\,{\cot }^{2}{\theta }_{0}+1)$ as shown by Fig. 3(a), it is sufficient to approximate $(4\,{\cot }^{2}{\theta }_{0}+1)\cong {\alpha }_{0}{({\mu }_{0}H)}^{-1/4}$ for the range of the experimental *θ*_0_ (~30°), leading to

3 $${\theta }_{0}={\rm{acot}}\,[\sqrt{\{{\alpha }_{0}{({\mu }_{0}H)}^{-1/4}-1\}/4}]{\rm{.}}$$

The solid line in Fig. 2(b) shows the numerical evaluation of Eq. (3). Though the experimental data appears scattered in comparison to the small variation of *θ*_0_, the solid line accords well with the experimental data. Please note that Eq. (3) does not contain any fitting parameters, because *α*_0_ was determined from an independent measurement.

From the creep criticality, one can find a logarithmic dependence $\alpha ({H}_{x}){({\mu }_{0}{H}_{z})}^{-1/4}=\,\mathrm{ln}({V}_{0}/{V}_{{\rm{DW}}})$, where *V*_0_ is the characteristic DW speed and *α* is the creep scaling constant. Therefore, Eq. (3) can be rewritten as ${\theta }_{0}={\rm{a}}{\rm{c}}{\rm{o}}{\rm{t}}[\sqrt{\{{\rm{l}}{\rm{n}}({V}_{0}/{V}_{{\rm{D}}{\rm{W}}})-1\}/4}]$. Due to the logarithmic dependence, *θ*_0_ is basically a slowly-varying function of *V*_DW_. Please note that, even if *V*_DW_ varies by 10 times, *θ*_0_ changes by only a few degrees, as we discuss later. For various samples with different *V*_0_, one may have to measure *V*_0_ for each sample to exactly estimate *θ*_0_. However, *θ*_0_ is less sensitive to *V*_0_ and also, the measurement of *V*_0_ takes about 1 hour for each sample, which is significantly easier and faster than the direct measurement of the angle dependence (taking more than 5 hours). Once the measurement of *V*_0_ is done, *θ*_0_ can be estimated for any desired *V*_DW_.

### Two-Dimensional Contour Map of Bubble Motion with respect to *θ* and *H*

To further check the validity of the present theory, we measure the two-dimensional contour map of $v\,(\theta ,H)$, which is plotted with respect to *θ* and $H$ as shown by Fig. 3(b). The colour contrast is scaled with the value of $\mathrm{log}(v)$ as the scale bar shown on the right lower end. For this plot, $v$ was experimentally measured for each values of *θ* and $H$ over the range of *θ* from 10 to 65° with 5° step and the range of ${\mu }_{0}H$ from 16 to 24 mT with 1-mT step. In the map, each colour traces each equi-speed contour. Several equi-speed contours are highlighted by the circular symbols, of which the position $(\theta ,H)$ indicates the values of *θ* and $H$ of the same speed for each equi-speed contour. The purple solid lines plot the prediction from the present model. It is clear from the figure that the model prediction matches well with the experimental results, confirming the validity of the present theory.

### **Dependence of***θ*_0_**on***H***and*α***_0_

**Figure 4:**
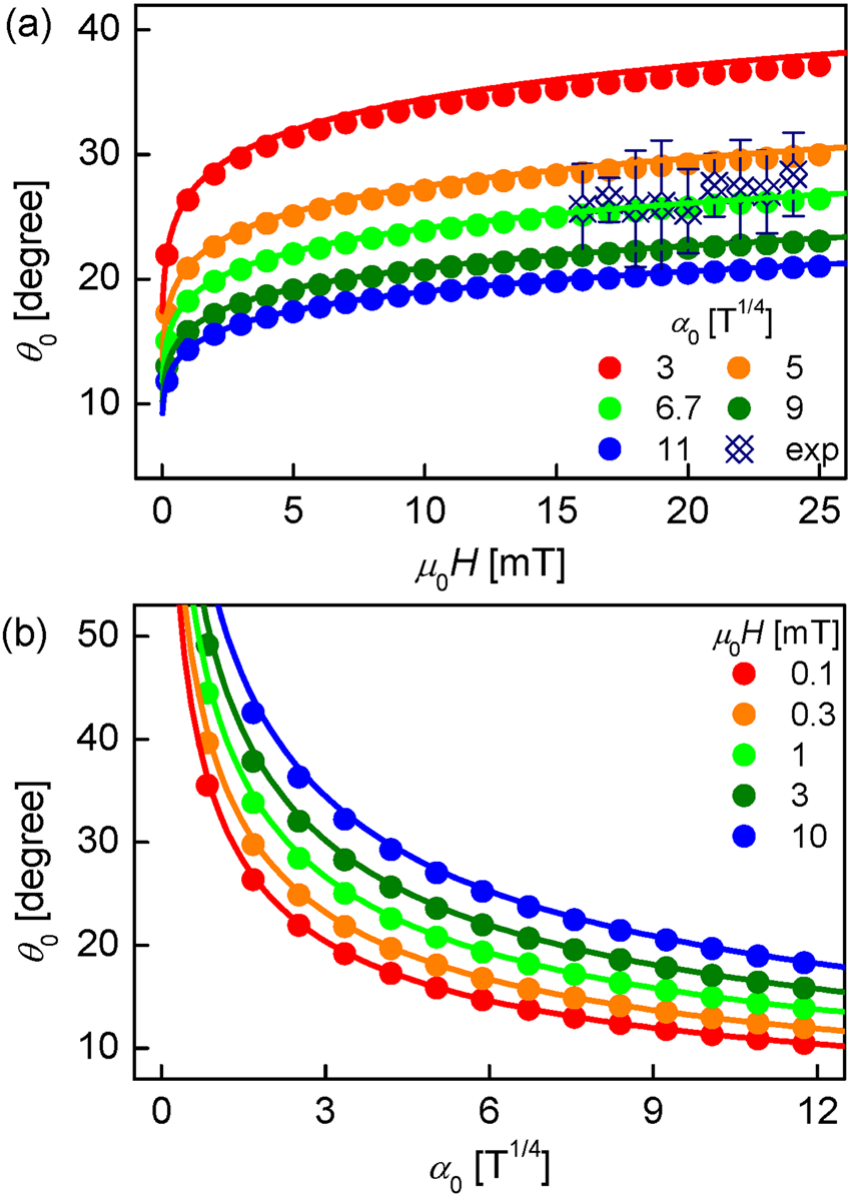
The dependence of *θ*_0_ as a function of *H* and *α*_0_. (**a**) *θ*_0_ with respect to *H* for various *α*_0_ and (**b**) *θ*_0_ with respect to *α*_0_ for various *H*. The circular symbols are obtained by solving Eq. (2) numerically and the solid lines are from Eq. (3). The open symbols in (**a**) indicate the experimental data with the error bars are obtained by the root-mean-square error of the parabolic fitting.

Figure 4 examines the dependence of *θ*_0_ on *H* and *α*_0_. The circular symbols are obtained by solving Eq. (2) numerically and the solid lines are from Eq. (3). The good conformity between the symbols and lines verifies again the validity of the approximation used to obtain Eq. (3). Figure 4(a) plots *θ*_0_ with respect to *H* for several fixed *α*_0_ over the practical range for Pt/Co/Pt films^13, 15, 20, 21, 25, 26^. The figure shows that, for all the values of *α*_0_, *θ*_0_ increases drastically as *H* increases up to about 3 mT and then, exhibits a slow variation as *H* increases further. Figure 4(b) plots *θ*_0_ with respect to *α*_0_ for several fixed *H*. It is also seen that *θ*_0_ is greatly reduced for the range of small *α*_0_, but slow variation for the range of large *α*_0_. The present observations provide a general guideline for the optimal *θ*_0_ to be about 30° for practical experimental conditions.

## Discussion

We would like to mention that $v$ can be affected by the asymmetries caused by other mechanisms such as chiral damping^16, 27, 28^ or DW width variation^29^. Since the DW width has a dependence on *H* and *β* is proportional to the DW width, the value of *β* also varies with respect to *H*. However, it is confirmed for the present films that the DW width variation is small (<30%)^30^ and that the chiral damping can be ignored owing to the experimental observation of parabolic $v$ dependence on ${H}_{x}$ ^15^. The good conformity of the present model to the experimental results reciprocally verifies that the asymmetry of the present films is mainly governed by the DW energy variation and thus, the present films provide a good test system to examine the magnetic bubblecade caused by the DMI-induced asymmetries. Other films with large effects from the other mechanisms^26–29, 31–33^ require further investigation for each optimal configuration.

We remark that the bubblecade can be realized even in the depinning and flow regimes, where the speed is much faster than one of the creep regime^25^. A similar DW-speed asymmetry appears in those regimes^16^. Though the nature of the DW-speed asymmetry in the depinning and flow regimes is not fully understood yet, the typical asymmetric behavior is almost the same with the creep regime. It is worthwhile to note that the bubblecade speed is given by $\,v\cong {\gamma }_{1}{H}_{x}$, where ${\gamma }_{1}$ is the slope of the DW speed asymmetry at ${H}_{x}=0$. Therefore, due to the similar DW-speed asymmetry, it has been demonstrated that a high-speed bubblecade can be also achieved^9^. Therefore, a similar behaviour of the optimal angle is expected even in the depinning and flow regimes, though the exact value of the optimal angle might be a little bit changed. An exact formula of *θ*_0_ can be available by the present approach, once the analytic formula on ${H}_{x}$ dependence is uncovered in the depinning and flow regimes.

It is technologically challenging to achieve small bubbles and their bubblecade motion for higher data storage capability. In this study, we could squeeze the bubbles down to a few μm in size as shown by Fig. 1(b). We expect that, eventually, the present operation principle has to be applied to the skyrmions, which also show the motion driven under oscillating magnetic field^17^. The bubblecade is generated by repeated process of expanding and shrinking bubble and thus, it has a frequency independence of the alternating magnetic field^9^. On the other hand, the skyrmions have a distinct frequency dependence due to the limited amount of expansion and shrinkage in size. The skyrmion speed can be maximized at the resonance modes in the high frequency regime^17, 34^. All these motions basically follows the same operation principles and therefore, we believe that the present study provides the knowledge and technological step toward the skyrmions-based bubblecade motion and devices.

The maximum applicable strength of the magnetic field is limited inevitably by the domain nucleation field of the sample and therefore, the maximum bubblecade speed is also limited. The critical field for nucleation varies largely among samples. This critical field is related to the stability of the data storage and process devices. In the last few decades, enormous efforts have devoted to achieve highly stable magnetic films and structures for achieving magnetic devices^6–9^. As one of the potential candidates, the present Pt/Co/Pt films exhibit a pretty good characteristics with the nucleation field up to a few hundred mT and also, the DW speed faster than a few tens or hundreds m/s without nucleation.

The applicable range of the alternating frequency is limited by the bubble annihilation process, since a longer duration of the magnetic field possibly gives the chance for the bubble to be collapsed even under application of a small magnetic field. Therefore, for practical implementation, one has to adjust both the frequency and amplitude of the alternating magnetic field to prevent the annihilation.

In conclusion, we examined the optimal configuration of the external magnetic field for the magnetic bubblecade. From the clear angular dependence of the bubblecade speed, the optimal angle for the maximum speed was determined experimentally and explained theoretically by a model based on the DW creep theory. The optimal angle is finally given by a simple equation of the amplitude of the alternating magnetic field. Our findings directly elucidate the major factors on the dynamics in the magnetic bubblecade, enabling the design of the optimal device configuration.

## Methods

### Sample preparations

For this study, Pt/Co/Pt films with strong perpendicular magnetic anisotropy (PMA) were prepared^20^. The detailed layer structure is 5.0-nm Ta/2.5-nm Pt/0.3-nm Co/1.0-nm Pt, which was deposited on a Si wafer with 100-nm SiO_2_ by use of dc magnetron sputtering. All the films exhibit clear circular domain expansion with weak pinning strength^9, 20^. This film has sizeable DMI, which induces asymmetric DW motion.

### Measurement of the bubble speed

The magnetic domain images were observed by use of a MOKE microscope equipped with a charge-coupled device camera. To apply a tilted magnetic field onto the films, a Ferris-wheel-like electromagnet is mounted to the microscope, such that it revolves on the *x*-*z* plane around the focal point of the microscope. The magnetic field can be varied up to 35 mT on the focal plane. The tilt angle of the electromagnet can be controlled from 0 to 90° in 5° steps. To measure the bubblecade speed $v$, a magnetic bubble was initially created by use of the thermomagnetic writing technique^9, 14, 21^. To apply alternating magnetic field, a magnetic field pulse of +*H* with a duration time $\Delta t$ is applied with an angle *θ* and successively, a reversed magnetic field pulses of −*H* with the same $\Delta t$ and *θ* is applied. To eliminate pulse-induced saturated magnetization or nucleation of new bubble, the proper condition of $\Delta t$ can be considered. After application of each field pulse, the domain image is captured by the MOKE microscope. The bubblecade speed is calculated by measuring the center position of the bubble in each image.
